# Supplementary material for: A Mac-2 Binding Protein Glycosylation Isomer-Based Risk Model Predicts Hepatocellular Carcinoma in HBV-Related Cirrhotic Patients on Antiviral Therapy
Source: Cancers (Basel). 2022 Oct 16;14(20):5063. doi: 10.3390/cancers14205063 (PMC9599873; doi:10.3390/cancers14205063)
Supplement: Supplementary file 1 [file cancers-14-05063-s001.zip › cancers-1935963-supplementary.pdf]

Supplementary Table S1. Baseline characteristics in the development and validation groups

Variables	Development group<br>n = 668	Validation group<br>n = 335	p Value
Age (year)	54.0 ± 11.7	53.8 ± 12.0	0.807
Sex, male	500 (79.6%)	246 (73.4%)	0.628
HBeAg-positive status	166 (24.8%)	81 (24.2%)	0.816
Decompensation status	134 (26.8%)	55 (17.1%)	0.164
NA-naïve	567 (83.6%)	282 (84.9%)	0.771
Diabetes mellitus, yes	150 (24.6%)	71 (21.5%)	0.649
Hypertension, yes	181 (33.3%)	82 (24.6%)	0.374
HBV DNA, $\log_{10}$ IU/mL	5.40 ± 1.53	5.48 ± 1.46	0.412
AST, U/L	130.1 ± 266.3	125.1 ± 242.0	0.771
ALT, U/L	164.3 ± 371.3	143.2 ± 289.0	0.364
Total bilirubin, mg/dL	2.20 ± 4.20	1.82 ± 2.70	0.054
Albumin, g/dL	3.98 ± 0.64	3.98 ± 0.63	0.999
INR	1.20 ± 0.29	1.19 ± 0.21	0.641
Platelet, $\times 10^3/\mu L$	135.1 ± 56.0	136.2 ± 57.2	0.772
AFP, ng/mL	27.9 ± 83.4	36.0 ± 165.4	0.302
M2BPGi, COI	3.04 ± 3.70	2.80 ± 3.42	0.313
HBcrAg, $\log_{10}$ U/mL	5.12 ± 1.51	5.11 ± 1.45	0.904

Abbreviations: AFP, alpha-fetoprotein; ALT, alanine aminotransferase; AST, aspartate aminotransferase; COI, cut-off index; HBcrAg, hepatitis B core related antigen; HBeAg, hepatitis B e antigen; HBV, hepatitis B virus; INR, international normalized ratio; M2BPGi, Mac-2 binding protein glycosylation isomer; NA, nucleos(t)ide analogu

Supplementary Table S2. Total risk scores predict the rates of 2–10 year hepatocellular carcinoma in the development cohort

Total scores	Year 2	Year 3	Year 4	Year 5	Year 6	Year 7	Year 8	Year 9	Year 10
**0**	1.7%	2.5%	3.4%	4.1%	4.7%	5.6%	6.8%	7.9%	7.9%
**1**	2.5%	3.8%	5.2%	6.2%	7.1%	8.5%	10.2%	11.8%	11.8%
**1.5**	3.1%	4.6%	6.3%	7.6%	8.7%	10.4%	12.4%	14.4%	14.4%
**2**	3.9%	5.7%	7.8%	9.3%	10.7%	12.7%	15.1%	17.5%	17.5%
**2.5**	4.8%	7.0%	9.5%	11.4%	13.0%	15.5%	18.4%	21.2%	21.2%
**3**	5.9%	8.6%	11.7%	14.0%	15.8%	18.8%	22.2%	25.6%	25.6%
**3.5**	7.2%	10.6%	14.2%	17.0%	19.2%	22.7%	26.7%	30.6%	30.6%
**4**	8.8%	12.9%	17.3%	20.6%	23.2%	27.3%	32.0%	36.4%	36.4%
**4.5**	10.8%	15.7%	21.0%	24.8%	27.9%	32.6%	37.9%	42.9%	42.9%
**5**	13.2%	19.1%	25.3%	29.7%	33.3%	38.6%	44.6%	50.0%	50.0%
**5.5**	16.1%	23.0%	30.3%	35.4%	39.4%	45.3%	51.8%	57.6%	57.6%
**6**	19.5%	27.7%	36.0%	41.7%	46.2%	52.6%	59.5%	65.4%	65.4%
**6.5**	23.6%	33.0%	42.4%	48.8%	53.6%	60.3%	67.3%	73.1%	73.1%

**7**	28.3%	39.1%	49.5%	56.3%	61.3%	68.2%	75.0%	80.4%	80.4%
**7.5**	33.7%	45.9%	57.1%	64.1%	69.2%	75.7%	82.0%	86.7%	86.7%
**8**	39.9%	53.2%	64.9%	71.9%	76.7%	82.7%	88.0%	91.7%	91.7%
**8.5**	46.8%	61.0%	72.7%	79.2%	83.5%	88.6%	92.8%	95.4%	95.4%
**9**	54.2%	68.8%	79.9%	85.7%	89.2%	93.2%	96.1%	97.8%	97.8%
**9.5**	61.9%	76.3%	86.3%	91.0%	93.7%	96.4%	98.2%	99.1%	99.1%
**10**	69.8%	83.2%	91.4%	94.9%	96.7%	98.4%	99.3%	99.7%	99.7%
**10.5**	77.2%	89.0%	95.2%	97.5%	98.5%	99.4%	99.8%	99.9%	99.9%
**11**	84.0%	93.4%	97.7%	99.0%	99.5%	99.8%	99.9%	99.9%	99.9%
**11.5**	89.6%	96.6%	99.0%	99.6%	99.8%	99.8%	99.9%	99.9%	99.9%

Supplementary Table S3. *P* values of AUROC comparisons between the ASPAM-B score and each risk score in the development group

Years	APAB versus ASPAM-B	PAGE-B versus ASPAM-B	RWS-HCC versus ASPAM-B	AASL-HCC versus ASPAM-B	THRI versus ASPAM-B
3	0.0044	0.0201	0.0005	0.0189	0.0029
5	0.0222	0.0419	0.0006	0.0041	0.0087
7	0.0228	0.0274	0.0004	0.0027	0.0021
9	0.0253	0.0445	0.0012	0.0055	0.0071

Abbreviation: AUROC, the area under the receiver operating characteristic curve.
